# Determination of ***β***-Cyano-L-alanine, ***γ***-Glutamyl-***β***-cyano-L-alanine, and Common Free Amino Acids in *Vicia sativa* (Fabaceae) Seeds by Reversed-Phase High-Performance Liquid Chromatography

**DOI:** 10.1155/2014/409089

**Published:** 2014-12-22

**Authors:** Cristina Megías, Isabel Cortés-Giraldo, Julio Girón-Calle, Javier Vioque, Manuel Alaiz

**Affiliations:** Instituto de la Grasa (C.S.I.C.), Avenida Padre García Tejero 4, 41012 Sevilla, Spain

## Abstract

A method for determination of *β*-cyano-L-alanine, *γ*-glutamyl-*β*-cyano-L-alanine and other free amino acids in *Vicia sativa* is presented. Seed extracts were derivatized by reaction with diethyl ethoxymethylenemalonate and analyzed by reverse-phase high-performance liquid chromatography. Calibration curves showed very good linearity of the response. The limit of detection and quantification was 0.15 and 0.50 *μ*M, respectively. The method has high intra- (RSD = 0.28–0.31%) and interrepeatability (RSD = 2.76–3.08%) and remarkable accuracy with a 99% recovery in spiked samples. The method is very easy to carry out and allows for ready analysis of large number of samples using very basic HPLC equipment because the derivatized samples are very stable and have very good chromatographic properties. The method has been applied to the determination of *γ*-glutamyl-*β*-cyano-L-alanine, *β*-cyano-L-alanine, and common free amino acids in eight wild populations of *V. sativa* from southwestern Spain.

## 1. Introduction

*Vicia sativa* is a forage legume best adapted to the semiarid regions of the Mediterranean basin and Australia [1]. Although the seeds of* V. sativa* are rich in protein [2], their use as animal feed and for human consumption is very limited.* V. sativa* can be highly toxic to mammals due to the presence of the heat stable neurotoxin dipeptide *γ*-glutamyl-*β*-cyano-L-alanine (GCA) and to a lesser extent to the presence of the related amino acid *β*-cyano-L-alanine (BCA) [3]. High levels of BCA and GCA in the diet of monogastric animals can result in respiratory difficulty, muscular and neurological alterations, and convulsion prior to death. The concentration of BCA in* V. sativa* seeds varies from 0.10% to 0.97% [4].* V. sativa* also accumulates GCA in the seeds at concentrations ranging from 0.41 to 1.36% [5].

There are few methods for determination of BCA and GCA in* V. sativa* seeds. These include quantification by diffuse reflectance infrared spectrometry [6] and two high-performance liquid chromatography (HPLC) methods [7, 8]. The goal of this research was to determine whether HPLC chromatography of the ethoxymethylenemalonate (DEEMM) derivatives of free amino acids can be used for determination of BCA and GCA as well as other free amino acids in the seeds of* V. sativa*. DEEMM is a universal reagent for amino groups and has been used in amino sugar [9] and amino acid [10] chemistry, as well as for amino acid analysis [11–17].

## 2. Materials and Methods

### 2.1. Plant Material

Seeds were collected from eight* V. sativa *populations at the Sierra de Aracena y Picos de Aroche Natural Park, in Huelva province (Spain). The GPS data for the eight locations were as follows: sample 1, N 37.896518, W 6.558431; sample 2, N 37.846477, W 6.473732; sample 3, N 37.890240, W 6.608969; sample 4, N 37.905338, W 6.616766; sample 5, N 37.918874, W 6.665784; sample 6, N 37.917389, W 6.667023; sample 7, N 37.902965, W 6.67023; sample 8 N 37.894393, W 6.709989. Seeds (30 g) were ground using a MM 301 mill (Retsch, Haan, Germany).

### 2.2. Reagents

DEEMM, BCA, dl-2-aminobutyric acid (internal standard, I.S.), amino acid standards, water (HPLC grade), and acetonitrile (HPLC grade) were purchased from Sigma-Aldrich (St. Louis, MO, USA). *γ*-Glutamyl-*β*-cyano-L-alanine was purified from* V. sativa *as described [18].

### 2.3. Chromatographic System

The HPLC system (Beckman-Coulter) consisted of a 126 solvent module, 166 detector, and IBM personal computer. Data acquisition and processing were carried out using 32 Karat 7.0 version software (Beckman-Coulter). Samples (20 *μ*L) were injected in a Nova Pak C18, 300 × 3.9 mm i.d., and 4 *μ*m reversed-phase column (Waters), and elution was carried out at 0.9 mL/min using a 25 mM glacial acetic acid/acetonitrile binary gradient as shown in Table 1. Mobile phases were filtered through a 0.45 *μ*m membrane filter. The column was maintained at 18°C.

### 2.4. Preparation of Sample

Samples (2.0 mg) were stirred in ethanol : water (3 : 7 v/v, 1 mL) for 30 min at room temperature and centrifuged at 12000 rpm for 10 min. Pellets were reextracted twice more, and the resulting supernatants were pooled and taken to dryness under nitrogen.

### 2.5. Precolumn Derivatization

Internal standard (24 *μ*L, 0.424 g/L) and DEEMM (2 *μ*L) were added to the samples in 1 M borate buffer pH 9.0 (3 mL). The solution was thoroughly mixed and incubated at 50°C for 50 min. Samples were filtered through 0.22 *μ*m membranes before injection into the HPLC system (20 *μ*L).

### 2.6. Evaluation of the Method

Evaluation was carried out by determination of linearity, limit of detection (LOD), limit of quantification (LOQ), repeatability, and accuracy (recovery) [19]. Calibration curves were drawn by plotting the peak area ratio of analyte/internal standard against reference analyte concentrations (determined in triplicate). LOD is the lowest concentration of analyte that is detectable by an analytical method and LOQ is the lowest solute concentration that can be determined with acceptable precision and accuracy. LOD and LOQ were calculated by injecting diluted standard solutions to determine the concentrations corresponding to a signal/noise ratio (*S/N*) of 3 and 10, respectively. The repeatability of the method was determined by the same analyst from the relative standard deviation (RSD) of the peak area based on 8 runs of a solution of the standard over 1 day (intraday repeatability) and from the RSD of the peak area based on 8 runs of a solution of the standard on independent days (interday repeatability). Accuracy was tested by the standard procedure of adding three increasing concentrations of BCA and GCA stock solution (10, 30, and 90 *μ*M) to a seed flour sample. Nonspiked sample replicates (blanks) were used to determine the initial BCA and GCA contents of the seed. The percentage recovery at each concentration was calculated as [(amount found in the sample spiked sample) − (amount found in the blank)/(amount added)] × 100.

### 2.7. Statistical Analysis

The RSD was calculated according to the formula RSD = *s*/*μ* × 100, where *s* is the standard deviation and *μ* is the average value. It was expressed as a percentage. The Microsoft Office Excel 2003 data analysis package was used for statistical analysis.

## 3. Results and Discussion

Like other* Vicia* species, the seeds of* Vicia sativa* contain numerous antinutritional factors, notably the cyanogenic amino acids BCA and GCA, and cyanogenic glycosides that are toxic to monogastric animals.* Vicia sativa* has been implicated in numerous cases of intoxication leading to stock losses. Its use for feeding pigs and poultry (the latter being the most sensitive) and for human nutrition is therefore restricted [4]. Unprocessed* Vicia sativa* seeds at 60% (w/w) in the diet were detrimental to chicken, causing 100% mortality in broilers with an average survival time of 5.1 days [20]. The high toxicity of BCA and GCA highlights the importance of methods that allow for a fast and reliable quantification of these seed components.

**Figure 1:**
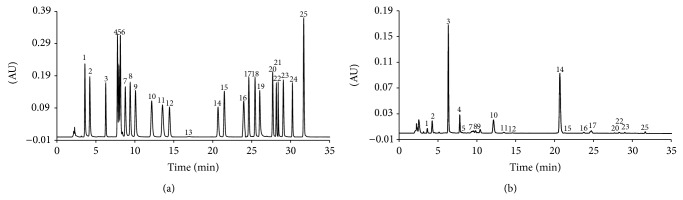
HPLC analysis of the DEEMM derivatives of GCA, BCA, and amino acid standards (a) and seed extracts (b). 1 = Asp; 2 = Glu; 3 = GCA; 4 = Asn; 5 = Ser; 6 = Gln; 7 = His; 8 = Gly; 9 = Thr; 10 = Arg; 11 = Ala; 12 = BCA; 13 = Pro; 14 = I.S.; 15 = Tyr; 16 = ammonium ion; 17 = Val; 18 = Met; 19 = Cys-Cys; 20 = Ile; 21 = Trp; 22 = Leu; 23 = Phe; 24 = Cys; 25 = Lys.

Precolumn derivatization of BCA, GCA, and standard amino acids by reaction with DEEMM resulted in stable derivatives with a very good chromatographic behavior in reversed-phase HPLC. These derivatives were readily detected at 280 nm with low detection limits and with no interference from the reagent. Most of these components were identified in the* V. sativa* extracts by comparison with authentic standards (Figure 1(a)). The DEEMM derivative of BCA and GCA eluted at 14.48 and 6.20 min, respectively, and their peaks did not overlap with any other amino acid. GCA was the major amino compound in the seeds (Figure 1(b)).

Analysis of 0.50 to 200 *μ*M BCA and GCA showed linear response (*r* ^2^ > 0.999), low LOD (0.15 *μ*M), and low LOQ (0.50 *μ*M) (Table 2). Peak areas for derivatized BCA and GCA were essentially unchanged for at least one week at room temperature as indicated by the low interday repeatability (RSD = 2.76–3.08%). Thus, the BCA and GCA DEEMM derivatives are stable enough to allow for storage for several days at room temperature before analysis. The intraday repeatability with an RSD below 0.30% was excellent. The accuracy of the method is also supported by the recovery of BCA and GCA from seed extracts to which 10, 30, or 90 *μ*M BCA and GCA were added. About 99% recovery (RSD = 0.20–0.76%) was possible after extraction and derivatization (Table 3).

This method was applied to the determination of BCA and GCA in the seeds of eight* V. sativa* populations at the Sierra de Aracena y Picos de Aroche Natural Park, in Huelva province (Spain) (Table 4). Contents of BCA and GCA ranged from 0.003 to 0.022 g/100 g and from 0.572 to 1.252 g/100 g, respectively. The values obtained for GCA in all populations are within the range described by other researchers [5]. GCA was the major free amino acid in* V. sativa* samples, representing from 44 to 79% (w/w) total free amino acids. The analysis also showed much lower amounts of the other common amino acids (Table 4).

As compared to other HPLC methods previously reported for determination of BCA and GCA [7, 8], the major advantages of this method are its simplicity and the stability of reagents and derivatized amino compounds. This allows for accurate, easy determination of BCA and GCA in a large number of samples using unsophisticated equipment such as a basic HPLC system with an UV detector.

## 4. Conclusions

Reverse phase HPLC of DEEMM derivatives allows for determination of BCA, GCA, and other free amino acids in the seeds of* V. sativa*. As compared to other methods, this procedure has a number of advantages: it is easy to carry out in any standard HPLC device, does not use any toxic reagent, and can be used to easily process a high number of samples because the derivatized amino acids are stable even at room temperature. The analysis is based on the very good chromatographic and absorption characteristics of the DEEMM derivatives, which allow for very good resolution of the peaks, as well as very good sensitivity and repeatability.

## Figures and Tables

**Table 1:** Chromatographic gradient conditions for the analysis of GCA, BCA, and free amino acids.

Time (min)	Eluent A^a^	Eluent B^b^
0	96	4
3	88	12
13	88	12
30	69	31
35	69	31
40	96	4

^a^25 mM glacial acetic acid, 0.02% (w/v) sodium azide pH 6.0.

^b^Acetonitrile.

**Table 2:** Precision, calibration parameters, and sensitivity of the determination of GCA and BCA by HPLC.

Compound	Repeatability (%)		Calibration			LOQ^d^ (*µ*M)	LOD^e^ (*µ*M)
	Intraday^a^	Interday^b^	Regression equation	*r* ^2^	Linear range^c^ (*µ*M)		
GCA	0.28	2.76	*y* = 33.52*x* + 0.0877	0.9998	0.50–200	0.50	0.15
BCA	0.31	3.08	*y* = 33.25*x* + 0.0971	0.9997	0.50–200	0.50	0.15

^a^RSD of peak area based on 8 runs of a solution of the standard over 1 day.

^b^RSD of peak area based on 8 runs of a solution of the standard on independent days.

^c^Concentration range between the limit of quantification and the upper linear limit.

^d^Limit of quantification: signal/noise ratio = 10.

^e^Limit of detection: signal/noise ratio = 3.

*y* = concentration (*µ*M).

*x* = peak area analyte/peak area internal standard.

**Table 3:** Recovery (mean and RSD) of the HPLC method for determination of GCA and BCA in *V. sativa* (sample 7 of Table 4).

Compound	Initial content (*μ*M)	First concentration added (10 *μ*M)		Second concentration added (30 *μ*M)		Third concentration added (90 *μ*M)	
		Content I (*µ*M)	Recovery	Content II (*µ*M)	Recovery	Content III (*µ*M)	Recovery
GCA	37.83	47.81	99.83 (0.76)	67.82	99.97 (0.16)	127.67	99.82 (0.33)
BCA	0.61	10.58	99.67 (0.55)	30.56	99.82 (0.20)	90.53	99.91 (0.09)

**Table 4:** Contents (% w/w of dry weight) of GCA, BCA, and free amino acids in *V. sativa* seeds. Data are the mean of three determinations ± standard deviation.

Compound/sample	1	2	3	4	5	6	7	8
GCA	0.887 ± 0.008	1.071 ± 0.021	0.572 ± 0.015	1.029 ± 0.031	1.134 ± 0.020	0.946 ± 0.027	1.252 ± 0.018	1.010 ± 0.075
BCA	0.003 ± 0.000	0.005 ± 0.000	0.006 ± 0.000	0.017 ± 0.001	0.022 ± 0.001	0.007 ± 0.001	0.009 ± 0.000	0.003 ± 0.000
Asp	0.094 ± 0.002	0.029 ± 0.002	0.016 ± 0.001	0.013 ± 0.001	0.029 ± 0.000	0.013 ± 0.001	0.031 ± 0.002	0.027 ± 0.001
Glu	0.036 ± 0.000	0.058 ± 0.002	0.081 ± 0.002	0.083 ± 0.003	0.082 ± 0.005	0.040 ± 0.001	0.099 ± 0.003	0.066 ± 0.000
Asn	0.010 ± 0.001	0.031 ± 0.003	0.189 ± 0.003	0.045 ± 0.002	0.045 ± 0.000	0.021 ± 0.002	0.048 ± 0.004	0.046 ± 0.002
Ser	0.010 ± 0.001	0.007 ± 0.000	0.006 ± 0.000	0.005 ± 0.000	0.006 ± 0.000	0.005 ± 0.000	0.007 ± 0.001	0.006 ± 0.000
Gln	0.000 ± 0.000	0.000 ± 0.000	0.000 ± 0.000	0.000 ± 0.000	0.000 ± 0.000	0.000 ± 0.000	0.000 ± 0.000	0.000 ± 0.000
His	0.009 ± 0.001	0.006 ± 0.000	0.037 ± 0.001	0.003 ± 0.000	0.002 ± 0.000	0.014 ± 0.001	0.013 ± 0.002	0.013 ± 0.001
Gly	0.009 ± 0.000	0.007 ± 0.000	0.007 ± 0.000	0.006 ± 0.001	0.006 ± 0.000	0.001 ± 0.000	0.005 ± 0.001	0.002 ± 0.000
Thr	0.065 ± 0.005	0.140 ± 0.003	0.014 ± 0.001	0.071 ± 0.003	0.028 ± 0.002	0.005 ± 0.001	0.007 ± 0.001	0.012 ± 0.001
Arg	0.072 ± 0.001	0.098 ± 0.009	0.301 ± 0.012	0.074 ± 0.012	0.157 ± 0.017	0.059 ± 0.002	0.124 ± 0.006	0.026 ± 0.003
Ala	0.007 ± 0.001	0.008 ± 0.001	0.014 ± 0.001	0.009 ± 0.001	0.014 ± 0.001	0.010 ± 0.001	0.012 ± 0.002	0.010 ± 0.001
Pro	0.000 ± 0.000	0.000 ± 0.000	0.000 ± 0.000	0.000 ± 0.000	0.000 ± 0.000	0.000 ± 0.000	0.000 ± 0.000	0.000 ± 0.000
Try	0.003 ± 0.000	0.002 ± 0.000	0.000 ± 0.000	0.000 ± 0.000	0.002 ± 0.000	0.002 ± 0.000	0.003 ± 0.001	0.002 ± 0.000
Val	0.009 ± 0.001	0.007 ± 0.001	0.007 ± 0.000	0.013 ± 0.001	0.004 ± 0.000	0.003 ± 0.000	0.003 ± 0.001	0.004 ± 0.001
Met	0.000 ± 0.000	0.000 ± 0.000	0.000 ± 0.000	0.000 ± 0.000	0.000 ± 0.000	0.000 ± 0.000	0.000 ± 0.000	0.000 ± 0.000
Cys-Cys	0.000 ± 0.000	0.000 ± 0.000	0.000 ± 0.000	0.000 ± 0.000	0.000 ± 0.000	0.000 ± 0.000	0.000 ± 0.000	0.000 ± 0.000
Ile	0.047 ± 0.001	0.002 ± 0.000	0.005 ± 0.000	0.005 ± 0.000	0.000 ± 0.000	0.001 ± 0.000	0.009 ± 0.000	0.009 ± 0.001
Trp	0.000 ± 0.000	0.000 ± 0.000	0.000 ± 0.000	0.000 ± 0.000	0.000 ± 0.000	0.000 ± 0.000	0.000 ± 0.000	0.000 ± 0.000
Leu	0.011 ± 0.001	0.014 ± 0.000	0.020 ± 0.001	0.007 ± 0.000	0.011 ± 0.000	0.002 ± 0.000	0.007 ± 0.000	0.019 ± 0.001
Phe	0.003 ± 0.000	0.003 ± 0.000	0.007 ± 0.001	0.005 ± 0.000	0.007 ± 0.000	0.008 ± 0.000	0.004 ± 0.000	0.008 ± 0.000
Cys	0.000 ± 0.000	0.000 ± 0.000	0.000 ± 0.000	0.000 ± 0.000	0.000 ± 0.000	0.000 ± 0.000	0.000 ± 0.000	0.000 ± 0.000
Lys	0.003 ± 0.000	0.003 ± 0.000	0.004 ± 0.001	0.003 ± 0.000	0.004 ± 0.000	0.004 ± 0.001	0.004 ± 0.000	0.002 ± 0.000
